# An Overview of Repurposed Drugs for Potential COVID-19 Treatment

**DOI:** 10.3390/antibiotics11121678

**Published:** 2022-11-22

**Authors:** Kamini Govender, Anil Chuturgoon

**Affiliations:** Discipline of Medical Biochemistry, School of Laboratory Medicine and Medical Sciences, College of Health Sciences, University of KwaZulu-Natal, Durban 4013, South Africa

**Keywords:** COVID-19, repurposing of drugs

## Abstract

The COVID-19 pandemic caused by SARS-CoV-2 has placed severe constraints on healthcare systems around the globe. The SARS-CoV-2 virus has caused upheaval in the healthcare and economic sectors worldwide. On the 20th of May 2020, the World Health Organisation declared COVID-19 a global pandemic due to the unprecedented number of cases reported around the globe. As of the 4th of November 2022, there were 637,117,429 coronavirus cases reported globally by Worldometer stats, with 6,602,572 related deaths. In South Africa, there were approximately 4,029,496 coronavirus cases and 102,311 associated deaths. As such, there is a need for efficacious therapeutic regimes. There has been a paucity of knowledge encompassing the use of effective and specific antiviral drug therapies for treating COVID-19 since the outbreak. In this review, we provide valuable insights into the repurposing of current drugs for COVID-19. Drug repurposing provides a suitable option for the discovery of efficacious drugs for COVID-19, thereby decreasing the costs and turnaround times of drug development strategies. This review provides an overview of ten drugs, including antimalarial, antiparasitic, anti-inflammatory, nucleoside analogue, monoclonal-antibody drugs, that were repurposed for the potential treatment of COVID-19.

## 1. Introduction

**Figure 1:**
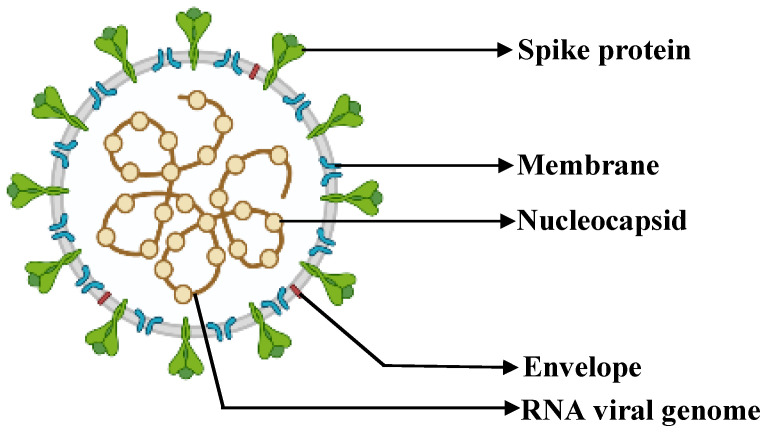
Illustration of the viral structure of SARS-CoV-2. Adapted from BioRender [8].

COVID-19 was first discovered in 2019 in Wuhan, China [1,2,3,4]. The disease is caused by severe acute respiratory syndrome coronavirus 2 (SARS-CoV-2), which belongs to the family *Coronaviridae* [5,6,7], genus *Betacoronavirus,* and subgenus *Sarbecovirus* [1,5]. The viral structure of SARS-CoV-2 comprises structural proteins, such as spike, membrane, nucleocapsid, and envelope proteins (Figure 1).

There is a paucity of knowledge pertaining to the treatment therapies for COVID-19 and to antiviral drugs [9]. The rapid spread of COVID-19 has resulted in unprecedented challenges, with millions of deaths and severe repercussions for economies and health worldwide [1,10]. Currently, the measures in place for controlling the spread of COVID-19 are supportive and preventative measures, such as quarantining and self-isolation [10,11,12].

The emergence of variants of SARS-CoV-2 jeopardises the success of current vaccinations and COVID-19 therapies worldwide [13,14,15,16]. The rollout of the COVID-19 vaccine has progressed significantly and has decreased the rate of mortality worldwide, but the emergence of the omicron variant in 2021 has resulted in uncertain times [17].The emergence of new COVID-19 variants has exacerbated the need for drug treatment regimens [18]. Due to the re-emerging viral infections, effective drug strategies are currently not available [19].

There has been a paucity of knowledge encompassing the use of drugs to treat COVID-19 since its outbreak. There is a severe need for effective antiviral drug treatments for COVID-19. There are currently no gold-standard drug therapy regimes in place for the treatment of COVID-19, which exacerbates the increasing rate of mortality and overall cases worldwide [9]. Drug development and clinical evaluations are not easy tasks and can take up to several years to develop from the proof of concept to production [9,20]. In drug discovery, it takes approximately 10–12 years for the development of a drug from proof of concept to commercialisation. However, new drug therapy approaches can take longer—up to 15 years [20]. There is a need for antiviral drugs that display high specificity for and efficacy against COVID-19. Due to the long turnaround times for the development of new drugs, a lucrative COVID-19 treatment strategy for decreasing the current burden on healthcare systems is the repurposing of known/existing drugs. Drug repurposing can be implemented as an effective strategy, firstly, in order to shorten delivery times, as well as to lower production costs [21]. De novo drug development strategies are estimated to cost approximately USD 1 billion [22].

As such, this review focuses on the repurposing of ten existing anti-inflammatory, antimalarial, and antiparasitic drugs, as well as nucleoside analogues and monoclonal antibodies. In this review paper, we conducted an extensive search in the available online literature on anti-inflammatory, antimalarial, and antiparasitic drugs, as well as nucleoside analogues and monoclonal antibodies, while taking the adverse side effects and the FDA recommendations into consideration. The keyword search criteria included: COVID-19, chloroquine, hydroxychloroquine, ivermectin, ebselen, remdesivir, molnupiravir, favipiravir, bebtelovimab, sotrovimab, and crizanlizumab. Furthermore, we decided to proceed with the ten aforementioned drugs for the repurposing of possible COVID-19 treatments.

A review conducted by Hossen et al. (2020) stated that the U.S. FDA and National Institutes for Health (NIH) recommended the use of a nucleoside analogue, remdesivir, as it displays promising potential for the treatment of SARS-CoV-2 [9]. According to Valle et al. (2020) there were approximately 2400 clinical trials reported; however, they stated that the drugs tested were limited to 20. It should be noted that their mechanisms of action require further investigation [23]. As such, the aforementioned authors also stated that the swiftest approach to drug therapy for COVID-19 would be the repurposing of existing drugs that are currently in the market and used for other diseases [23].

## 2. Repurposing of Drugs for Treatment of COVID-19

### 2.1. Antimalarial Drugs

#### Chloroquine and Hydroxychloroquine

**Figure 2:**
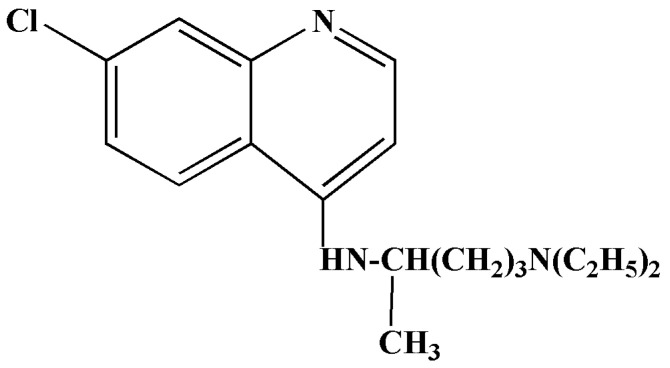
Structure of chloroquine [26].

Chloroquine is an antimalarial drug that displays broad-spectrum antiviral properties [24,25]. Chloroquine is classified as a 4-aminoquinoline compound (Figure 2), and it is employed to treat diseases such as malaria, rheumatoid arthritis and autoimmune diseases such as lupus erythematosus [21,26,27,28]. It is also a weak base, and the precise mode of action has not been elucidated for all microorganisms. However, there are two key proposed mechanisms of action for chloroquine; the first occurs in the vesicles of both fungi and bacteria, and this is the alkalisation of the phagolysosome. This process inhibits viral replication, fusion, and uncoating, as these processes are dependent on a low-pH environment [27]. An in vitro study conducted by Wang et al. reported that chloroquine treatment was efficacious against SARS-CoV-2 with an effective concentration (EC_50_) of 1.13 μM [29]. 

**Figure 3:**
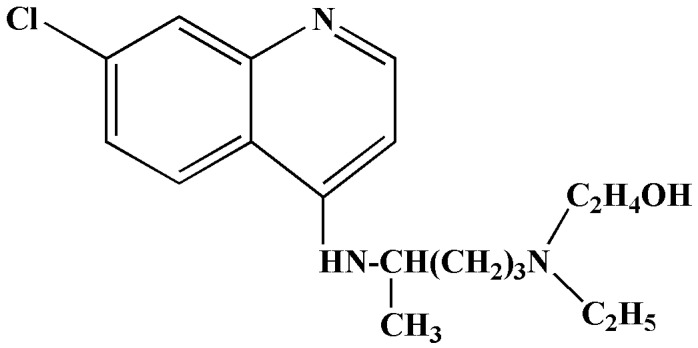
Structure of hydroxychloroquine [26].

Hydroxychloroquine is a structural analogue of chloroquine (Figure 3) [30,31]. The mode of action and activity are similar to those of chloroquine. Hydroxychloroquine contains a hydroxyl group and is less toxic, with a better safety profile [30,31]. It was previously used to treat diseases such as lupus, rheumatoid arthritis, and malaria, since it exhibits immunomodulatory activities [9,31,32,33,34]. A study conducted by Yao et al. (2020) tested hydroxychloroquine and chloroquine in vitro on the Vero cell line. The data indicated that hydroxychloroquine had an EC_50_ of 0.72 μM, whereas chloroquine had an EC_50_ of 5.47 μM [31]. The hydroxychloroquine drug was administered based on physiologically based pharmacokinetic (PBPK) models 400 mg twice per day, with a subsequent 200 mg double dose daily (4 days) [31].

The proposed mechanism of action is that the drug alters the pH on the cells’ surfaces, thereby inhibiting the virus from binding to the cell membranes of the host. It inhibits viral replication, release, and assembly, protein glycosylation of the virus, and the transportation of new viral particles [26]. Hydroxychloroquine inhibits the coronavirus via a series of steps; however, the exact molecular mechanism has not yet been elucidated [31].

Clinical trials are currently underway to assess the efficacy of hydroxychloroquine for the treatment of COVID-19 [30]. Since there is limited information regarding the efficacy and safety of the aforementioned drug in the treatment of SARS-CoV-2, patients and doctors worldwide should be informed about the potential risks, side effects, and benefits associated with their prescribed medications [35]. Some adverse side effects are retinopathy, hypoglycaemia, gastrointestinal discomfort, allergic reactions, and kidney, heart, and liver problems [33,34].

The FDA initially issued an EUA for hydroxychloroquine in the treatment of COVID-19 [30]. On the 15th of June 2020, the FDA revoked the EUA because the criteria in Section 564(c)(2) of the Food, Drug, and Cosmetic Act were not fulfilled, since there were adverse side effects that were related to adverse cardiac-related effects. Therefore, the risks outweighed the potential benefits and the EUA for chloroquine and hydroxychloroquine as effective treatment strategies for COVID-19 was revoked [36].

A meta-analytical review conducted by Deng et al. (2021) involved 61,221 hospitalised COVID-19 patients, and they concluded that they would not recommend continued or future treatment of hospitalised COVID-19 patients with chloroquine or hydroxychloroquine based on the lack of efficacy, since there were no significant reductions in the occurrence of mechanical ventilations, mortality, or length of stay in the hospital [37]. In another meta-analysis conducted by Di Stefano et al. (2022), the authors reiterated that chloroquine and hydroxychloroquine were not efficacious in the treatment of hospitalised COVID-19 patients [38]. However, another article published by Chen et al. (2021) stated that chloroquine and hydroxychloroquine had been involved in more than a hundred clinical trials [39]; once all of these trials were completed, a statement regarding efficacy could conclusively be made [40].

### 2.2. Antiparasitic Drugs

#### Ivermectin

Ivermectin—also referred to as Stromectol [9]—is derived from avermectin, which is produced by *Streptomyces avermitilis* [41]. Ivermectin is classified as a broad-spectrum antiparasitic drug [42,43], and it was previously utilised in the treatment of diseases such as river blindness and lymphatic filariasis, as well as in the treatment of parasitic worm infections [41,43]. Its mechanism of action in parasites affects the gamma-amino butyric acid (GABA) neurotransmitters by attaching to their glutamate chloride channels [43]. It is an FDA-approved drug [12]. Ivermectin is considered a wonder drug because it has a range of therapeutic uses, since it displays anti-cancer, anti-bacterial, and antiviral properties [44]. Ivermectin exhibits antiviral activities on RNA viruses such as dengue, Avian influenza A, and SARS-CoV-2 [44].

In an in vitro study conducted by Carly et al. (2020), the authors observed a 5000-time reduction in viral RNA after a 48 h treatment with ivermectin on the Vero/hSLAM cell line [12]. A review conducted by Heidary and Gharebaghi (2020) stated that in order for ivermectin to be efficacious, it should ideally be administered in the early stages of COVID-19 infection; however, human clinical trials are required [44].

The efficacy of ivermectin for the treatment of COVID-19 was reviewed by Izcovich et al. (2021); after a systematic review, they concluded that previous reports pertaining to the benefits of ivermectin were flawed, as there were methodological limitations. They also stated that ivermectin’s use as a prophylactic intervention and its effects on clinical improvement in COVID-19 patients were uncertain; therefore, they concluded that further research on ivermectin is required [45]. A report conducted by Schmith et al. (2020) concluded that the initial approved dose of 200 μg/kg of ivermectin was not adequate, since after oral administration, the predicted concentration eventually reaching the human lungs would not be efficacious in the treatment of COVID-19 (yielding low predicted lung concentrations of approximately 0.0873 μM). Therefore, they recommended the use of higher doses. However, this poses the risk of side effects, such as the outbreak of rashes, nausea, dizziness, and headaches [46]. The dosing (posology) and efficacy of ivermectin are highly controversial topics; therefore, more research needs to be conducted [47]. The ideal dose of ivermectin for the prophylaxis of COVID-19 was not successfully determined [46]. Chronic usage of ivermectin has also not been established [47]. There is also a paucity of knowledge encompassing the pharmacokinetic profile of ivermectin in humans [43]. The elucidation of the pharmacodynamics and pharmacokinetics of ivermectin with different dose responses is required in order to determine the effective dose in humans [46,48]. Therefore, further investigation is required for in vivo and clinical trials in humans in order to decipher the effects of the aforementioned drug on humans infected with COVID-19. Ivermectin is currently in phase 3 clinical trials with a 600 mcg dose. After these trials are completed, we can then make a conclusion regarding potential clinical management [49,50].

### 2.3. Anti-Inflammatory Drugs

#### Ebselen

**Figure 4:**
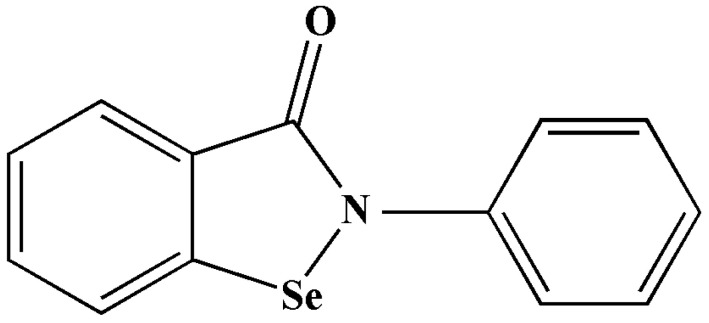
Illustration of the structure of ebselen [51,52].

Ebselen—also known as PZ-51, DR3305, and SPI-1005.—is categorised as a selenorganic drug (Figure 4), and it is capable of peroxynitrite and hydroperoxide activities [51,52,53]. This 2-phenyl-1,2-benzoisoselenazol-3(2H)-one compound was reported to have cytoprotective, anti-bacterial, anti-oxidant, and anti-inflammatory properties [53,54,55]. It also acts as an apoptosis inducer and a free-radical scavenger, and it is a neuroprotective agent [55]. It was identified as a mimetic glutathione peroxidase-1 and peroxiredoxin drug [52,53]. Ebselen was used to treat noise-induced hearing loss and bipolar mood disorder [56,57].

A study conducted by Jin et al. (2020) screened over 10,000 drugs with high-throughput methods in conjunction with structure-based virtual techniques to identify the potential main protease inhibitors of SARS-CoV-2. Their findings indicated that ebselen displayed promising antiviral activity with an EC_50_ of 4.67 μM [2].

SARS-CoV-2 encodes two cysteine proteases—first, the main protease, and then the papain-like protease [58]. These papain-like proteins have a role in viral replication (such as in the cleavage of viral proteins) and a role in the host immune response [58,59]. Ebselen was identified as a nonspecific cysteine modifier and a main and papain-like protease inhibitor [58,60]. However, in the presence of 1,4-dithiothreitol, protease inhibition was reduced or abolished [61]. Ma et al. concluded that ebselen is a non-specific protease inhibitor, and its mechanism of action is promiscuous, since other drug targets (nsp13 and nsp14) could be involved in the antiviral activities of SARS-CoV-2 [60].

This anti-inflammatory drug (ebselen) is currently in phase 2 clinical trials for the treatment of COVID-19 patients exhibiting mild to severe symptoms (NCT04483973 and NCT04484025) [55]. Further research needs to be conducted on ebselen, such as in vivo studies, as well as randomised clinical trials, in order to determine its efficacy as a drug treatment for COVID-19 [52].

### 2.4. Nucleoside Analogues

#### 2.4.1. Remdesivir

**Figure 5:**
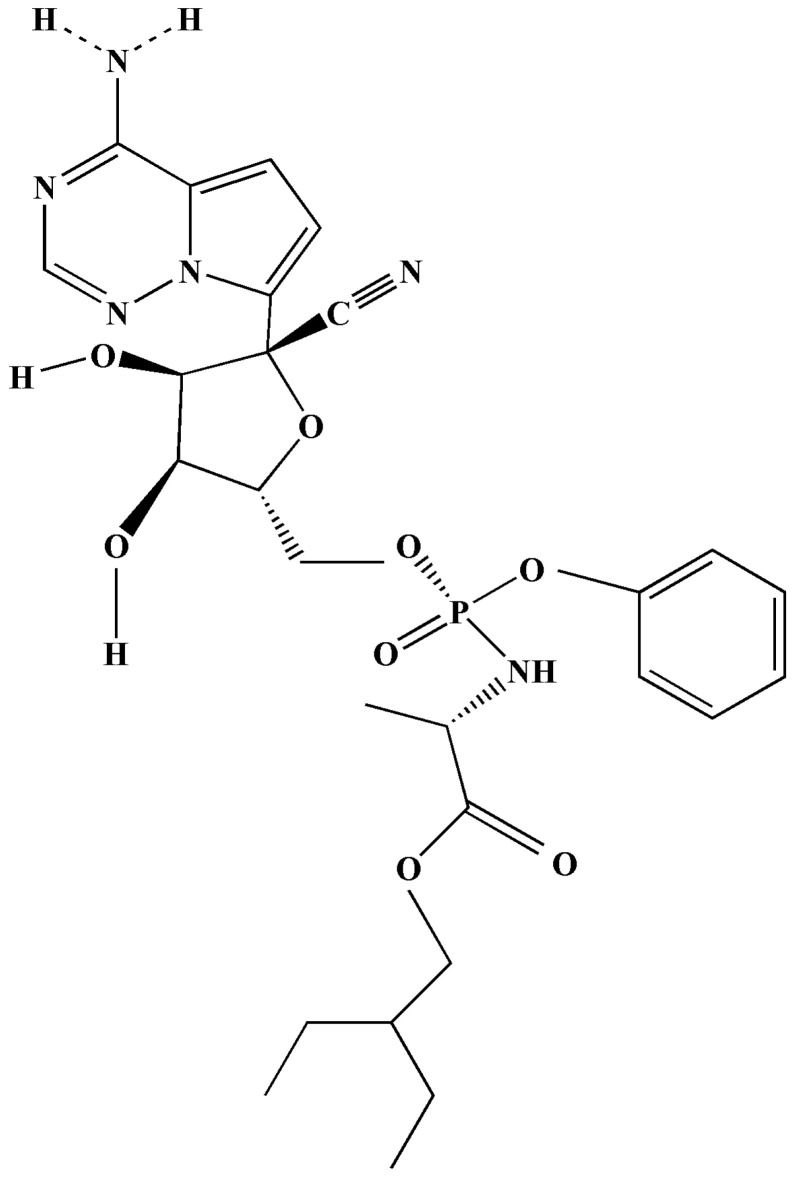
Depiction of the chemical structure of remdesivir (GS-5734) [63].

Remdesivir is classified as a nucleoside analogue (Figure 5) [9,62]. Remdesivir is also known as Veklury [63,64]. It should be noted that the aforementioned drug is not approved by the Food and Drug Administration. However, in 2020, they issued an emergency use authorisation (EUA) for patients suffering from severe COVID-19 symptoms [64].

**Figure 6:**
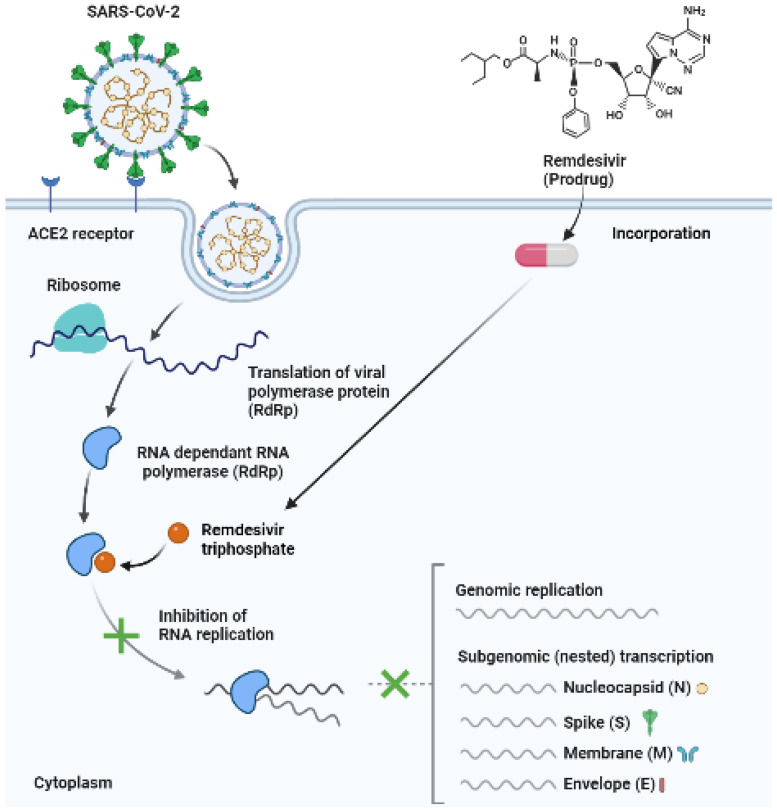
Illustration of the addition of remdesivir triphosphate, resulting in the inhibition of RNA replication and chain termination (open access); adapted from [66]. Created using BioRender [67].

According to Hossen et al. (2020), remdesivir is considered a promising drug for the treatment of COVID-19, since it has broad-spectrum antiviral activities in vitro against an array of RNA viruses, such as Pneumoviridae, Orthocoronavirinae, Filoviridae, and Paramyxoviridae [9]. The mode of action is the inhibition of RNA-dependent RNA polymerases, and it results in the early termination of RNA transcription, since it competes for inhibition with adenosine triphosphate (Figure 6) [29,65].

A clinical trial conducted by Beigel et al. (2020) revealed that COVID-19 patients had a decreased recovery of 10 days in comparison with that of the control group (placebo), which took 15 days [65]. Another study conducted by Grein et al, (2020) applied remdesivir for compassionate use in 53 COVID-19 patients; the oxygen support levels of 68% of these patients improved, but there were also side effects, such as liver dysfunction, renal impairment, diarrhoea, deep vein thrombosis, and septic shock [62]. Remdesivir was administered intravenously to the patients for 10 days, with an initial dose of 200 mg on day 1; 100 mg was given for the subsequent 9 days [62]. It should be noted that the sample size of the patients was small (n = 53) [62].

Remdesivir displayed antiviral activity in vitro in the Vero E6 cell line with an EC_50_ value of 23.15 μM [68]. Another study by Wang et al. (2020) reported that the aforementioned drug potently blocked SARS-CoV-2 infection with an EC_50_ of 0.77 μM [29]. 

In a paper by Young et al. (2021) that was published in *Lancet Infectious Diseases*, the authors stated that after reviewing a randomized phase 3, open-labelled clinical trial with 584 patients who experienced severe and moderate COVID-19 symptoms [1], the use of remdesivir did not offer a significant benefit after 28 days for the COVID-19 patients [69,70]. However, if remdesivir was administered early, i.e., less than ten days into the illness, it could shorten the recovery time and decrease the risk of progression of hyperinflammation [70]. However, the aforementioned clinical trial that was reviewed had limitations, such as the fact that the original protocol was confined to Asia, its open-label design resulted in a bias in the reported data and patient care, and the fact that remdesivir’s effect on the viral load of SARS-CoV-2 was not evaluated [69].

For the efficacy and safety of remdesivir, its lung penetration (tissue distribution) needs to be assessed, as the intravenous mode of administration in COVID-19 patients was not sufficient [71,72]. Larger multi-centre clinical trials are required to assess the efficacy, different routes of administration, and safety of remdesivir before it can be utilised for the treatment of COVID-19. Additionally, combinations of immune modulatory agents and antiviral therapies can be investigated for potential treatments for COVID-19 patients [72,73].

#### 2.4.2. Molnupiravir

**Figure 7:**
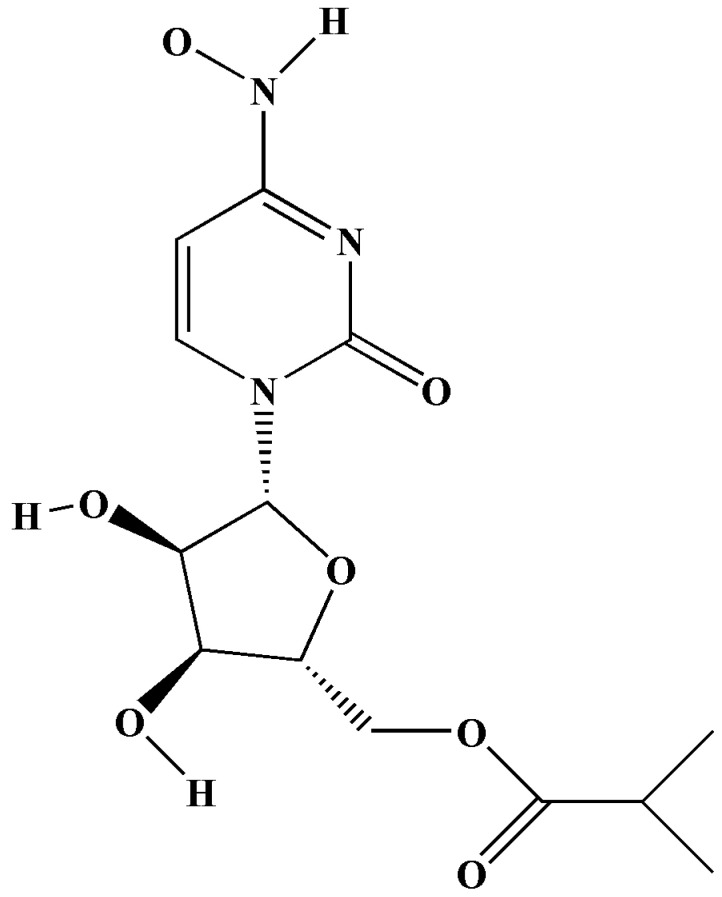
Structure of molnupirvir [18,80].

Molnupiravir—also referred to as MK-4482 and EIDD-2801 (Figure 7)—is another nucleoside analogue that has antiviral activities against a multiplicity of viruses, such as the SARS-CoV, ebola, MERS-CoV, SARS-CoV-2, and influenza viruses [18,74]. Molnupirvir is currently undergoing clinical trials for the treatment of COVID-19, and it exhibits anti-RNA polymerase activities [74].

Molnupiravir targets RNA-dependent RNA polymerase, which is important in the pathophysiology of COVID-19 and is a key enzyme in viral replication [74]. According to a review conducted by Pourkarim et al. (2021), molnupiravir is effective in the treatment of COVID-19 in animals; however, clinical trials are essential to the determination of its effect on COVID-19 patients [74]. The United Kingdom approved this antiviral first. Molnupiravir can be administered to patients suffering from mild to moderate COVID-19 symptoms and who may be at risk of developing a comorbidity, such as diabetes [75].

Molnupiravir decreases the replication of SARS-CoV-2, thereby impeding COVID-19 [21]. According to a review conducted by Zarenezhad and Marzi (2022) [76] and a perspective paper published by Lee et al. (2021) [77], it was indicated that clinical trials resulted in a decreased risk of fatality or hospitalisation in patients displaying mild to moderate COVID-19 symptoms [76,77].

Host esterases found in plasma convert molnupiravir into EIDD-193 [74]. A study conducted by Cox et al. (2021) involved a study of molnupiravir in ferrets (MK-4482/EIDD-2801). Prior to in vivo analyses, they tested the prodrug in vitro on the Vero E6 cell line, where it displayed an EC_50_ of 3.4 μM. According to a review article by Cox et al. (2021), molnupiravir is in advanced stages of clinical trials as a result of the successful completion of NCT04392219 (clinical trial phase 1) [78,79].

The use of molupiravir can provide the following benefits if taken during the early stages of infection: it can decrease local outbreaks, it can reduce the socioeconomic and emotional effects associated with increased isolation periods, it can reduce the progression of COVID-19, and it aids in speedy recovery [78]. The side effects associated with molnupiravir include influenza symptoms, diarrhoea, back pain, nausea, pain in the extremities, and headaches [74,79].

#### 2.4.3. Favipiravir

**Figure 8:**
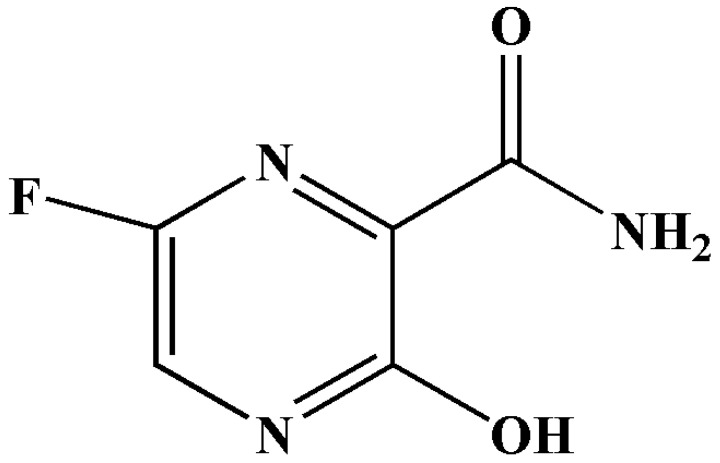
The structure of favipiravir [19].

Favipiravir (T-705)—also known as 6-fluoro-3-hydroxy-2-pyrazinecarboxamide—is classified as an antiviral. The aforementioned drug has a structure of a modified pyrazine analogue (Figure 8) [28]. It undergoes phosphorylation and ribosylation to provide an activated form of the drug (favipiravir-RTP) [28,81]. Essentially, this active metabolite is a nucleotide analogue and causes RNA-dependent RNA polymerase inhibition, thereby terminating viral replication in the SARS-CoV-2 genome via lethal mutagenesis and chain termination [28,82]. Favipiravir was initially developed for the treatment of influenza [19,28,81,83]. This prodrug displays a broad spectrum of antiviral properties in vitro [83], since the catalytic domain (influenza virus) is similar to those of other RNA viruses, such as SARS-CoV-2. According to a review conducted by Aherfi et al, (2021), the results of in vitro analyses were inconclusive [28]. There were two in vitro studies in which the Vero E6 cell line was treated with favipiravir; both concluded that the aforementioned drug was efficacious based on its cytopathic effects. However, they reported high EC_50_ values of 207 μM [82] and 204 μM [84] in the respective studies. However, clinical trials are underway in Italy, the United Kingdom, and China [82]. China approved the use of favipiravir for the treatment of SARS-CoV-2 in March 2020 [9].

An open-label clinical study observed the efficacy of favipiravir for the treatment of COVID-19 patients; on day one, a dose of 1600 mg was administered twice, followed by 600 mg twice daily for days 2–14. On the 14th day, there was a 91.43% improvement in the patients [85]. Another controlled study with 96 patients indicated that there was a reduction in hospitalisation and mechanical ventilation of COVID-19 patients [86].

It should also be noted that some of the side effects of using favipiravir are hyperuricemia and teratogenicity [81]. Therefore, it is not recommended that pregnant women use the drug, as it can cause birth defects or abnormalities in the embryo [21,81,85]. As such, more clinical testing is required to fully understand the pharmacokinetics, advantages, and disadvantages of the drug.

### 2.5. Monoclonal Antibodies (mAbs)

#### 2.5.1. Bebtelovimab

Bebtelovimab is classified as a recombinant monoclonal antibody (IgG1λ) [87]. Bebtelovimab is also known as LY-CoV1404 [15]. According to Westendorf et al. (2022), bebtelovimab displays highly potent neutralisation activities against the delta and omicron variants of SARS-CoV-2, and it exhibits a conserved epitope [15]. In vitro analyses were conducted in Vero E6 cell lines, and plaque reduction was measured and quantified with a dose–response model, with an EC_50_ value of approximately 6.4 ng/mL [87].

According to the FDA monoclonal antibodies such as bebtelovimabab displayed activity against the omicron variants BA.4/BA.5 and BA.2.12.1; as such, an EUA was issued in February 2022, since this drug can be used to treat COVID-19 patients who are adults and children who show mild to moderate symptoms (older than 12 years, with a minimum weight of forty kilograms) [87]. The dosage is 87.5 mg/mL [87].

**Figure 9:**
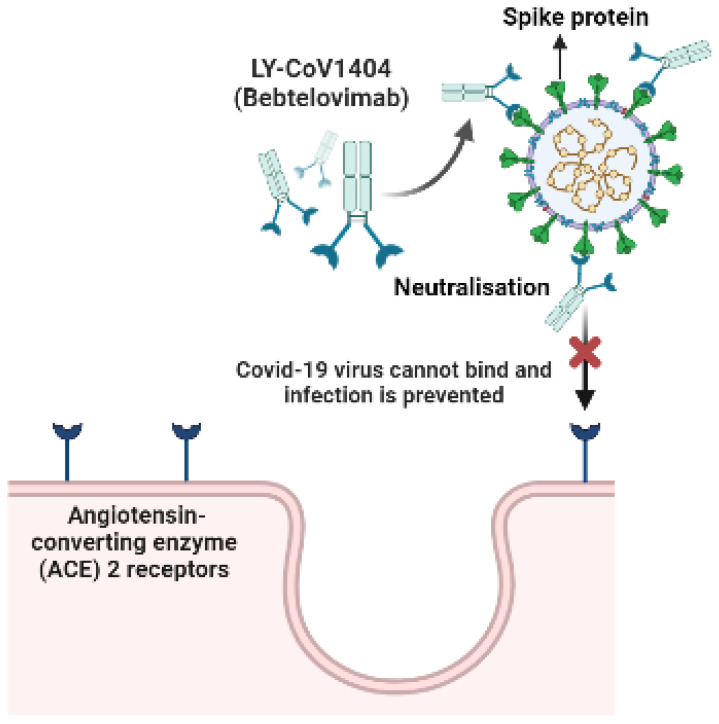
Neutralisation of the spike protein by bebtelovimab (open access); adapted from [15]. Created using BioRender [67].

The aforementioned drug binds to the spike protein of SARS-CoV-2 and inhibits the binding to the ACE2 receptor with an approximate dissociation constant of 0.046–0.075 nM (Figure 9) [87,88]. It has a half-maximal inhibitory concentration (IC50) of 0.39 nM. With regards to animal toxicology, according to the FDA, there were no severe effects on rats that were intravenously treated with bebtelovimab, and in cross-reactivity studies of human tissue, binding of clinical concern was not observed in the tissues [87]. Randomised phase 1 and 2 clinical trials are currently underway (BLAZE-4 trial (NCT04634409)) [87]. According to the FDA, the phase 2 and 3 data indicate that bebtelovimab can be utilised for the treatment of patients presenting with mild to moderate symptoms [87].

#### 2.5.2. Sotrovimab

Sotrovimab is also referred to as Xevudy^®^, and it is synthesised by Vir Biotechnology in conjunction with GlaxoSmithKline [89]. The FDA issued an EUA for the use of sotrovimab, which is a pan-sarbecovirus monoclonal antibody that is utilised in the treatment of patients exhibiting mild to moderate symptoms of COVID-19 or patients at risk for the rapid progression of the disease, such as those for whom this might result in hospitalisation or eventual fatality [89,90,91]. On the 17th of December 2021, it was fully approved in the European Union. It received its first full approval on 17 December 2021 [89].

There is a paucity of knowledge encompassing the clinical use of sotrovimab; adverse side effects, such as hypersensitivity and anaphylaxis, may occur, or infusion-related symptoms, such as dizziness, headache, fever, myalgia, arrhythmia, nausea, and difficulty breathing, might be experienced [90].

Sotrovimab should ideally be administered within seven days of a patient displaying COVID-19 symptoms, with a 500 mg dose via a single intravenous infusion [90]. The aforementioned drug is a recombinant human monoclonal antibody (IgG1-kappa); the mechanism of action is the binding of the drug to the receptor binding domain of the spike protein of SARS-CoV-2 (conserved epitope region), with a dissociation constant of approximately 0.21 nM [90]. The neutralisation activities of sotrovimab were tested in vitro against SARS-CoV-2 in the Vero E6 cell line, yielding an EC_50_ of 0.67 nM [90].

Clinical trials are currently underway; in a phase 3 double-blind placebo clinical trial (COMET-ICE, clinical trial government number: NCT04545060) with 583 patients as the sample size, the trial sites were located in four countries: Spain, the United States, Brazil, and Canada [91]. The findings of the aforementioned study indicated that sotrovimab decreased the risk of the disease progression of COVID-19 [91].

#### 2.5.3. Crizanlizumab

Crizanlizumab is a humanised monoclonal antibody [92]. Crizanlizumab is also referred to as SEG101 and Adakveo^®^; it was initially used for the treatment of sickle cell disease in patients aged 16 years and older [93]. The mechanism of action of crizanlizumab is its binding to P-selectin protein, thereby preventing the interaction with the P-selectin glycoprotein ligand 1 [92,93]. In severe cases of COVID-19, it is associated with thrombosis, elevated levels of P-selectin protein, and vascular inflammation [94]. In a placebo-controlled double-blind pilot clinical trial conducted by Leucker et al. (2021), 54 hospitalised COVID-19 patients displaying moderate symptoms were randomised [94]. A single dose of crizanlizumab was administered intravenously at 5.0 mg/kg; there was a reduction in P-selectin levels and an increase in D-dimer levels, and they concluded that a larger trial needs to be conducted [94]. According to McCarthy (2022), crizanlizumab showed therapeutic promise in a small cohort of COVID-19 patients [95]. ACTIV-4a provides a platform for testing various classes of repurposed drugs [95]. ACTIV-4a is a multicentre, open-label clinical trial study monitoring numerous targets involved in vascular homeostasis and the clotting cascades in COVID-19 patients [95]. The aforementioned monoclonal antibody is currently in ACTIV-4a continuous enrolment Q1 and phase 4 clinical trials for ACTIV-4a; the results will be available in 2023 for clinical management [49,50].

This review provides an overview of the drug repurposing for antimalarial, antiparasitic, anti-inflammatory, nucleoside analogue, and monoclonal-antibody drugs for the potential treatment of COVID-19 (refer to Table 1 and Table 2); however, the respective drugs have to undergo rigorous clinical trials in order to determine their efficacy and safety and to establish the effective dosages.

## 3. Conclusions

COVID-19 has caused unprecedented challenges in healthcare systems and a global socioeconomic upheaval. The COVID-19 pandemic has urged scientists around the globe to rapidly find effective treatment regimens for SARS-CoV-2. There are numerous factors to consider when developing new drugs, and drug repurposing is a viable option. The development of new drugs will cost billions; in order for us to combat the disease, scientists need to utilise drug-repurposing strategies to provide suitable drug therapies for COVID-19. Herein, we provided an overview of ten drugs that can potentially be employed to treat SARS-CoV-2.

Most reviews assess the use of antivirals, nucleoside analogues, and antimalarial drugs for the treatment of COVID-19. We reported the use of monoclonal antibodies (bebtelovimab and sotrovimab) that were highly efficacious thus far in the treatment of COVID-19. However, they are currently in phase 2 and 3 clinical trials. Clinical trials are underway to investigate the potential side effects and pharmacokinetics of chloroquine, hydroxychloroquine, ivermectin, ebselen, remdesivir, molnupiravir, and flavipiravir.

Currently, there is no effective approved clinical therapy for COVID-19 [73]. The time factor in drug discovery is a major limitation; since COVID-19 originated in 2019, large amounts of data are have resulted from tests in clinical trials. Generally, the approximate time for a drug for the treatment of a particular disease to go from proof of concept to commercialisation is 10–12 years. Therefore, it will be cumbersome to synthesise and develop new drugs for the treatment of COVID-19 rapidly in less than five years; as such, in this review, we reviewed the repurposing of ten existing drugs for the aforementioned therapy. In addition, the field of drug discovery is vast and rapid; as such, not all of the drugs used to treat COVID-19 can be discussed, since a new drug is being tested every day; future studies can elaborate further. This review provided a holistic overview of drug repurposing, and it included drugs such as remdesivir and monoclonal antibodies that, according to preliminary clinical trials, showed great promise for the treatment of COVID-19. It must be noted that we also included other drugs that were not as efficacious.

Future studies can employ molecular docking coupled with in vitro assays to aid in the discovery of potent drugs that can be repurposed for the treatment of COVID-19 [28]. In addition, research studies can also investigate the use of recombinant DNA methodologies to clone and express SARS-CoV-2 and purify these proteins via chromatography complemented with mass spectrometry.

## Figures and Tables

**Table 1:** A summary of the mechanisms of action of the ten drugs discussed in this review.

Drug	Mechanism of action	References
Chloroquine	Alkalisation of the phagolysosome; this process inhibits viral replication, fusion, and uncoating.	[27]
Hydroxychloroquine	Alters the pH on the cell surface, inhibiting the virus from binding to the cell membrane of the host. Inhibition of viral replication.	[26]
Ivermectin	Affects the GABA neurotransmitters by attaching to their glutamate chloride channels.	[43]
Ebselen	A mimetic glutathione peroxidase-1 and peroxiredoxin drug.	[52,53]
Remdesivir	Inhibition of RNA-dependent RNA polymerases; results in early termination of RNA transcription.	[29,65]
Molnupiravir	Targets RNA-dependent RNA polymerase.	[74]
Favipiravir	RNA-dependent RNA polymerase inhibition, terminating viral replication in the SARS-CoV-2 genome via lethal mutagenesis and chain termination.	[28,82]
Bebtelovimab	Binds to the spike protein of SARS-CoV-2 and inhibits the binding to the ACE2 receptor.	[87,88]
Sotrovimab	This drug binds to the SARC-CoV-2 spike protein’s receptor binding domain (conserved epitope region).	[90]
Crizanlizumab	Crizanlizumab binds to the P-selectin protein, thereby preventing the interaction with P-selectin glycoprotein ligand 1.	[92,93]

**Table 2:** A summary of the ten repurposed drugs for potential COVID-19 treatment that are under clinical trials.

Drug	Type of Drug	Clinical Trial	References
Chloroquine	Antimalarial	Phase 1, 2, and 3 clinical trials completed/withdrawn	[39]
Hydroxychloroquine	Antimalarial	Phase 3 clinical trials ended, withdrawn, terminated, and continuing.	[39]
Ivermectin	Antiparasitic	Phase 3 clinical trials	[49,50]
Ebselen	Anti-inflammatory	Phase 2 clinical trials	[96]
Remdesivir	Nucleoside analogue	Continuing phase 3 clinical trials	[49,50,97]
Molnupiravir	Nucleoside analogue	Phase 3 clinical trials	[98,99]
Favipiravir	Nucleoside analogue	Phase 2 and 3 clinical trials	[97]
Bebtelovimab	Monoclonal antibody	Phase 1, 2, and 3 clinical trials	[87]
Sotrovimab	Monoclonal antibody	Phase 3 clinical trials	[91]
Crizanlizumab	Monoclonal antibody	Phase 4 and continuous enrolment for Q1 2023	[49,50]
